# Evaluation of gastric submucosal tumors using endoscopically visualized features with submucosal endoscopy

**DOI:** 10.3892/ol.2014.2126

**Published:** 2014-05-08

**Authors:** HIDEKI KOBARA, HIROHITO MORI, KAZI RAFIQ, TAE MATSUNAGA, SHINTARO FUJIHARA, NORIKO NISHIYAMA, MAKI AYAKI, TATSUO YACHIDA, JOHJI TANI, HISAAKI MIYOSHI, KIYOHITO KATO, HIDEKI KAMADA, HIROHITO YONEYAMA, ASAHIRO MORISHITA, KUNIHIKO TSUTSUI, HISAKAZU IWAMA, REIJI HABA, TSUTOMU MASAKI

**Affiliations:** 1 Department of Gastroenterology and Neurology, Faculty of Medicine, Kagawa University, Miki, Kagawa 761-0793, Japan; 2 Department of Pharmacology, Faculty of Medicine, Kagawa University, Miki, Kagawa 761-0793, Japan; 3 Life Science Research Center, Faculty of Medicine, Kagawa University, Miki, Kagawa 761-0793, Japan; 4 Department of Diagnostic Pathology, Faculty of Medicine, Kagawa University, Miki, Kagawa 761-0793, Japan

**Keywords:** differential diagnosis, submucosal tumor, gastrointestinal stromal tumors, diagnostic techniques, endoscopic imaging, submucosal endoscopy

## Abstract

Although the macroscopic characteristics of submucosal tumors (SMTs), such as gastrointestinal stromal tumors (GISTs), have been characterized, the assessment of SMTs by their endoscopically visualized features (EVF; which are observed by endoscopic imaging under direct view) remains unevaluated. The aim of the present study was to investigate the potential of endoscopic diagnostics for SMTs using EVF. The EVF of 26 gastric SMT cases, in which the final pathological diagnosis was obtained by core biopsy using the submucosal endoscopy with mucosal flap method, were retrospectively reviewed. Each type of SMT was classified according to the following five EVF: Color, clarity, shape, tumor coating and solidity. Additionally, the EVF of 13 low-risk GISTs and 13 benign submucosal tumors (BSTs) were comparatively evaluated for the five abovementioned EVF. Similar trends were identified between the low-risk GISTs, granular cell tumors and the schwannoma with regard to EVF. However, while these tumors exhibited cloudy EVF, the leiomyomas tended to exhibit clear EVF. Among SMTs of the heterotopic pancreas type, the EVF demonstrated particularly small nodules of the pancreatic tissue itself. Although the sample size included in the present study is small, a classification system for gastric SMTs was proposed according to the EVF. When compared with the BST group, the GIST group demonstrated a significantly higher frequency of tumors that exhibited a combination of three EVF (white, cloudy and rigid) that are consistent with all gastric GISTs (P<0.05). Gastric SMTs may be classified based on the EVF, which indicates that the EVF possess potential diagnostic value for the differentiation of GISTs from BSTs.

## Introduction

The most common types of submucosal tumor (SMT) include mesenchymal tumors, such as gastrointestinal (GI) stromal tumors (STs), myogenic and neurogenic tumors, which collectively account for 54% of all SMT cases, followed in frequency by aberrant pancreases, cysts, lipomas, cartinoid tumors, lymphangiomas and hemangiomas (1). Among these SMTs, cases of GISTs are the most common. In 2004, the European Society for Medical Oncology Consensus GIST meeting declared that GISTs exhibit malignant potential and always require treatment by surgical resection (2). Therefore, it is clinically important to differentiate between GISTs and other types of SMT. The typical endoscopic characteristics of all SMTs include a lesion of hemispheric appearance with gently sloping edges that is covered by normal mucosa, however, these features do not aid with distinguishing between the histological types of SMT. Endoscopic ultrasound (EUS) is a key procedure in the evaluation of SMTs of the GI tract, as it enables determination of the layer of origin of the GI wall and allows for diagnostic sampling (3). However, differentiating between GISTs, leiomyomas and neurinomas using EUS is often complex as all of these tumors are visualized as a hypoechoic mass arising from the muscularis propria (MP), which is the typical EUS finding when observing mesenchymal tumors (4). Tissue sampling is therefore essential for obtaining an accurate diagnosis of SMTs.

Recently, EUS-guided fine needle aspiration has emerged as an important method for the diagnosis of SMTs. However, as this technique provides limited diagnostic accuracy due to the limited quantity of tissue sample that can be collected, an optimal method for tissue sampling is required (5,6). Endoscopic submucosal dissection (ESD), which involves the insertion of an endoscope into the submucosa (SM) to facilitate the dissection of the SM from the underlying muscle layer, enables an en bloc resection of early epithelial neoplasm and has become the standard approach for the resection of early GI cancer (7–10). The submucosal endoscopy with mucosal flap (SEMF) method (11) incorporating the ESD technique has been developed to permit a safer offset entry into the peritoneal cavity during natural orifice translumenal endoscopic surgery (12).

In our previous study, the value of core biopsy using the SEMF method was developed and demonstrated as a novel method for collecting tumor tissue under direct vision to assist in the diagnosis of SMTs (13,14). One technical advantage of core biopsy using the SEMF method is that once the ESD technique is complete, and upon creating a tunnel into the SM toward the tumor, the tumor can be visually identified, which enables the reliable collection of tumor tissue. Using this method, which provides direct visualization of the tumor, endoscopic images of the tumor can be obtained, which can be quantified for the macroscopic characteristics of SMTs, including the color, clarity and shape of the tumor surface, the presence or absence of a tumor capsule, and the solidity of the tumor (as assessed by pressure that is applied using forceps). Using closed forceps the mass can be probed to determine whether it is rigid, soft, or indents when depressed. The consistency of the mass can be symptomatic and aid with diagnosis. A mobile mass that is soft and indents when depressed using biopsy forceps is highly indicative of a benign tumor, such as a lipoma or a vascular or cystic tumor. By contrast, if a mass does not indent, it may indicate a firm lesion, such as a GIST or a leiomyoma. However, the specificity of these endoscopic characteristics has not been rigorously evaluated (1). A typical macroscopic GIST image is characterized as a multi-nodular, gray/white, hard tumor. Improved characterization of the endoscopic appearance of the surface of SMTs may further improve the diagnostic accuracy of core biopsies using the SEMF method combined with an endoscopic examination of the tumor surface.

## Patients and methods

### Patients

In total, 26 patients were enrolled in the present study (males, n=10 and females, n=16; mean age, 64.07 years; age range, 41–82 years) who were histologically diagnosed with gastric SMTs (GISTs, n=13; leiomyomas, n=5; granular cell tumors, n=2; heterotopic pancreases, n=2; cysts, n=2; schwannoma, n=1; and lipoma, n=1) by core biopsy using the SEMF method between November 2011 and October 2013 (Table I). All tumors were evaluated by routine EUS (20-MHz high-frequency miniprobe, UM-3R; Olympus Medical Systems, Tokyo, Japan) and computed tomography. SMTs originating from the SM or the MP were included and tumors presenting primarily with extra luminal growth were excluded, as such cases were considered to be high risk for perforation. The Clinical Ethics Committee of Kagawa University Hospital (Kagawa, Japan) approved the use of this procedure for gastric SMTs, and written informed consent was obtained from patients prior to the procedure.

### The SEMF method

**Figure 1:**
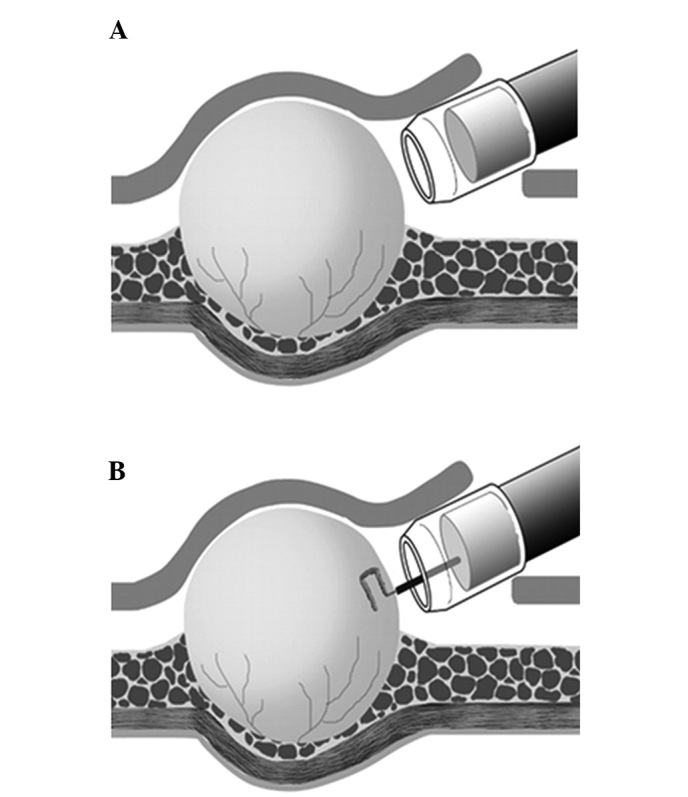
Bloc biopsy using the submucosal endoscopy mucosal flap (SEMF) method. (A) SEMF consisted of creating a short tunnel, via an additional submucosal dissection, to access the tumor. The characteristic endoscopically visualized feature findings of the submucosal tumors (SMTs) are shown in the submucosa from the dissected submucosal tunnel layer. (B) A bloc biopsy was performed to obtain a bloc specimen (size, 5×5×2 mm) using a needle-knife in the cutting mode of the electrosurgical unit. The hardness of the SMTs was assessed by applying pressure against the tumor using the needle-knife during the bloc biopsy.

The SEMF method consists of five major procedures as previously described (13,14): i) After demarcating the tumor borders with a margin of ~5 mm, a small incision is made to create a 10-mm opening flap (i.e. the ESD procedure). ii) A short tunnel, which is used to access the tumor, is made through the opening flap via an additional submucosal dissection (i.e. a short SEMF method). The tumor is visually identified and exposed (Fig. 1A). iii) A core specimen (5×5×2 mm) is obtained using a needle-knife (Olympus KD-441Q; Olympus Medical Systems) in the cutting mode provided by the electrosurgical unit (VIO 300D, EndoCut^®^ mode effect 2, duration 3; ERBE Elektromedizin GmbH, Tübingen, Germany) while minimizing compression of the tissue (i.e. a core biopsy; Fig. 1B). iv) The specimen is collected into a transparent cap that is designed to be longer at the tip (Elastic Touch F-01; TOP Corporation, Tokyo, Japan; i.e. the long attachment method for tissue collection), with care taken to prevent the tissue from coming into contact with the inner wall of the tunnel. v) The entire detached surface is sutured away from the periphery of the tumor with clips to prevent tumor fragments from flowing back into the tunnel (i.e. clip closure from the tumor side) and finally, a specimen that is sufficient for immunohistochemical analysis (~5-mm diameter), is obtained. Endoscopic images, still and moving, obtained for the 26 SMT patients during the second (Fig. 1A) and third (Fig. 1B) procedures were retrospectively reviewed. The short SEMF method (the second step) provides endoscopic visualization of the tumor under direct vision (endoscopically visualized features; EVF); these images may be quantified for the macroscopic characteristics of SMTs, including color, clarity, shape and presence or absence of a tumor capsule. The core biopsy (the third step) demonstrates the solidity of the tumor as assessed by pressure applied using closed forceps.

### Assessment I

**Figure 2:**
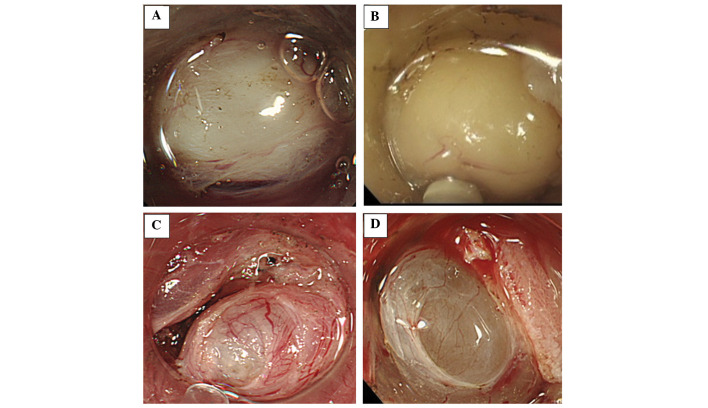
Typical endoscopically visualized feature (EVF) findings of submucosal tumors, with regard to color: White, yellow, blue or colorless. EVF findings of (A) a gastric gastrointestinal stromal tumor appearing as a white mass (case 3); (B) a lipoma with a yellow appearance (case 11); (C) a cyst with a blue appearance (case 13); and (D) a cyst with a colorless appearance (case 20).

**Figure 3:**
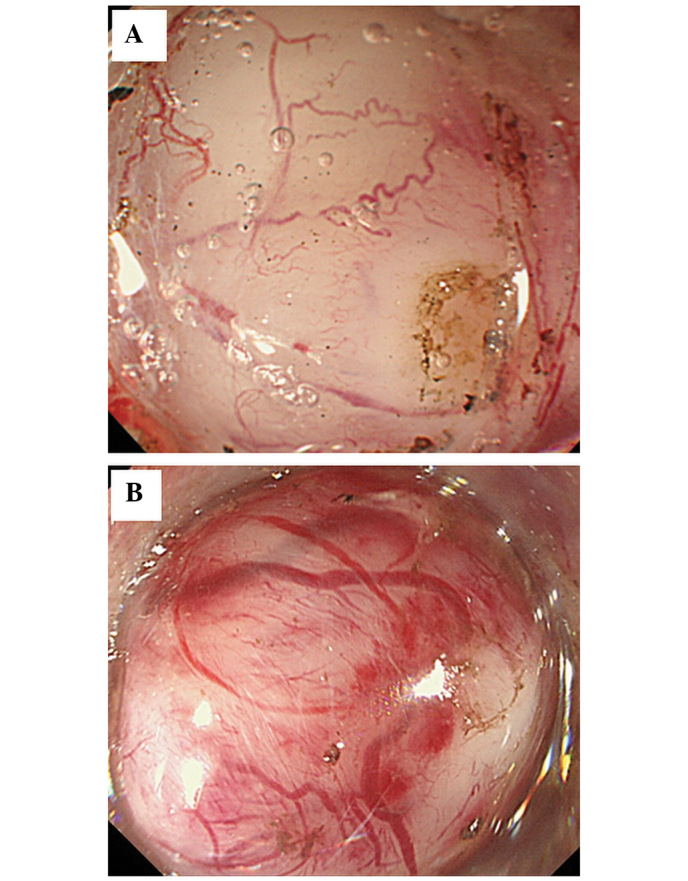
Typical endoscopically visualized feature (EVF) findings of submucosal tumors, with regard to the clarity of the tumor surface. The clarity was classified by two terms: Clear or cloudy. EVF findings of (A) a leiomyoma with a clear tumor surface (case 4) and (B) a gastrointestinal stromal tumor with a cloudy surface (case 23).

**Figure 4:**
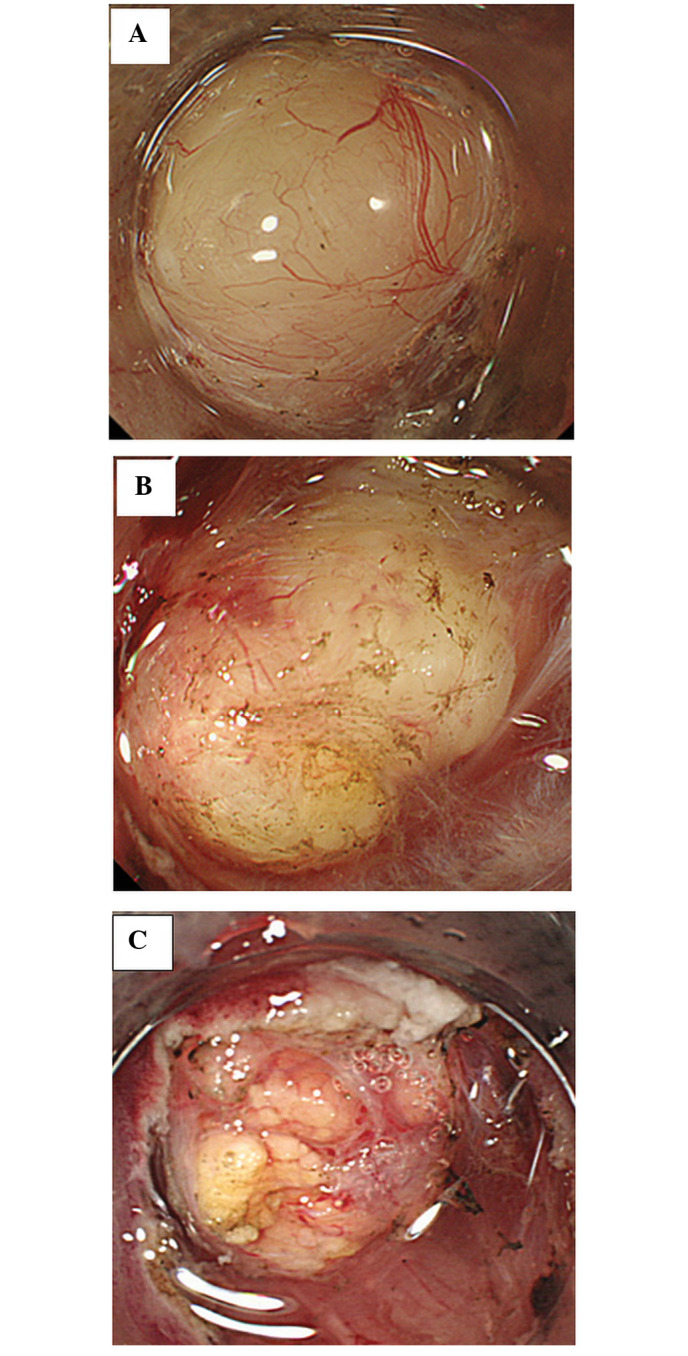
Typical endoscopically visualized feature (EVF) findings of submucosal tumors, with regard to the shape of the tumor surface. The shape was classified by two terms: Round or nodular. Additionally, nodular was subdivided to small or large nodular. EVF findings of (A) a leiomyoma with a round surface (case 26); (B) a gastrointestinal stromal tumor with a large nodular surface (case 22); and (C) a heterotopic pancreas with a small nodular surface (case 6).

**Figure 5:**
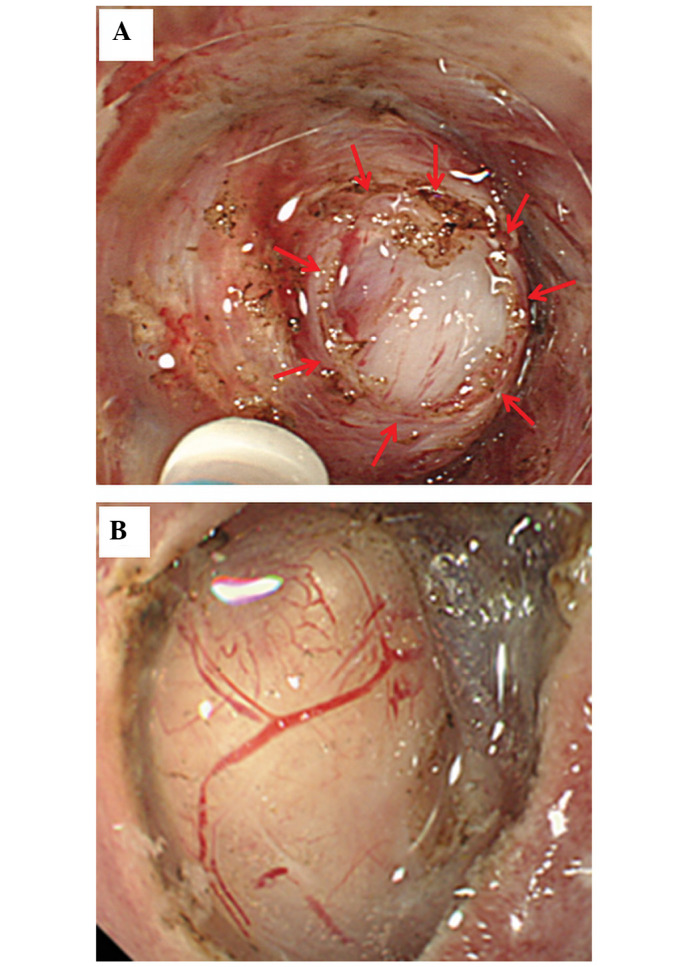
Typical endoscopically visualized feature (EVF) findings of submucosal tumors, with regard to the tumor coating. EVF findings of (A) a gastrointestinal stromal tumor with a visible capsule (red arrows indicate the perimeter of the capsule; case 7) and (B) a granular cell tumor where the capsule is not visible (case 18).

**Figure 6:**
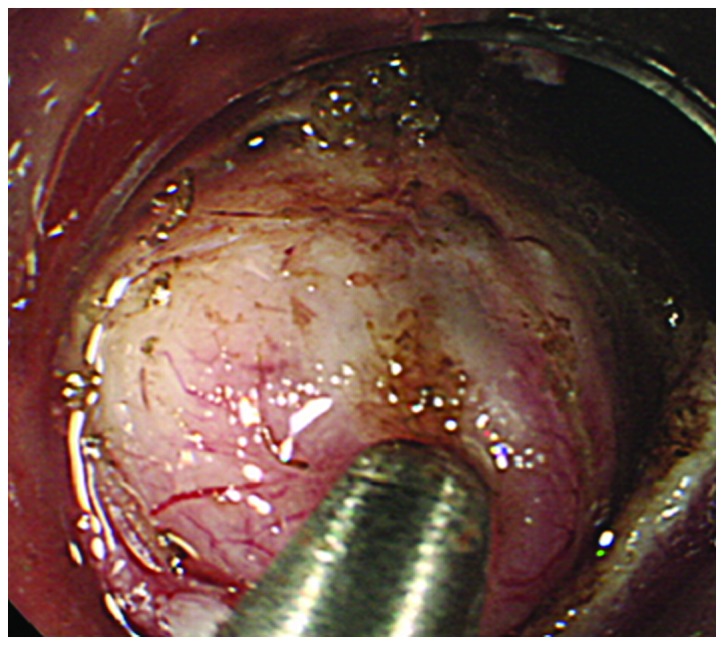
Typical endoscopically visualized feature (EVF) findings of submucosal tumors, with regard to rigidity. This GIST was rigid; it did not indent when compressed with a scalpel (case 9).

The five EVF for each type of SMT were evaluated and each type of SMT was classified based on these five EVF as follows: Color, clarity, shape, tumor coating and solidity. Colors were classified into four typical EVF colors: White, yellow, blue and colorless (Fig. 2A–D). The clarity was classified as either clear or cloudy (Fig. 3A and B). The shape was classified as either round or nodular (Fig. 4A–C). Additionally, the nodules were subdivided by size as small or large (Fig. 4B and C). The tumor coating was classified as either visible or not visible (Fig 5A and B). In addition, the solidity was classified as rigid (Fig. 6) or soft (Fig. 2B). Rigid tumors were defined as elastic and non-elastic hard tumors.

### Assessment II

In the retrospective comparative study, the EVF were compared between the 13 patients with gastric GISTs and the 13 patients with benign submucosal tumors (BSTs) with respect to color (white or not white), clarity, shape of the tumor surface, the presence or absence of a visible capsule and the rigidity (whether the mass indents when depressed) as evaluated by two endoscopists. Additionally, a combination of three EVF was compared between the two groups.

### Statistical analysis

The two-sided Fisher’s exact test was used for the comparison of the five tumor characteristics between the two groups. P<0.05 was considered to indicate a statistically significant difference.

## Results

### Assessment I

**Figure 7:**
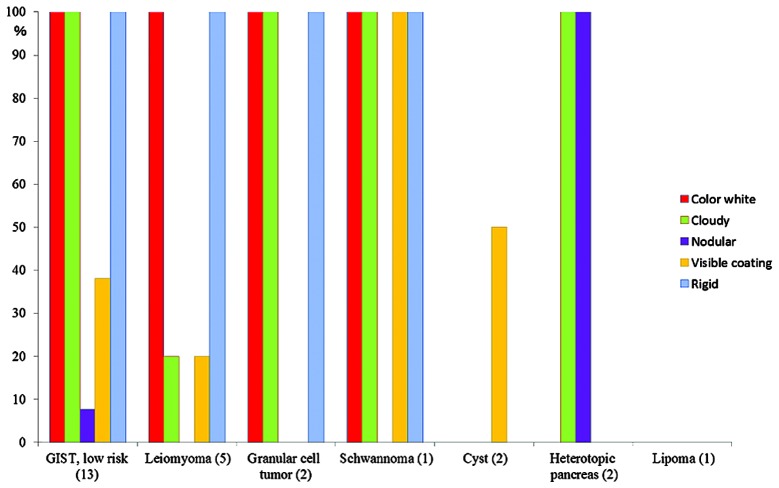
Percentages of each of the five endoscopically visualized features among gastric SMTs. Mesenchymal tumors, including 13 GISTs, five leiomyomas, two granular cell tumors and a single schwannoma, tended to have similar characteristics. Among the SMTs, heterotopic pancreases exhibited small nodules with the appearance of pancreatic tissue. GIST, gastrointestinal stromal tumor; SMT, submucosal tumor.

The EVF of SMTs in the individual cases are summarized in Table II. A histogram of the results was constructed to clearly demonstrate the differences between each SMT (Fig. 7). The mesenchymal tumors, including the 13 GISTs, five leiomyomas, two granular cell tumors and one schwannoma tended to exhibit similar characteristics. Among the SMTs, heterotopic pancreas revealed small nodules with an appearance similar to that of the pancreatic tissue itself or showing pancreatic-like tissue characteristics (Fig. 4C). A classification system of gastric SMTs using EVF is proposed on the basis of these results (Table III). The typical endoscopic findings of GISTs were tumors that were white, cloudy, round and rigid (Figs. 2A, 3B, 4B and 6). In the five cases of leiomyomas, the tumors were characterized as white, clear (n=4) > cloudy (n=1), round and elastic hard tumors (Figs. 3A and 4A). Although the sample size was small, two granular cell tumors (Fig. 5B) and one schwannoma were white, cloudy, round and rigid tumors, which is similar to GISTs. Conversely, in the two cases of gastric cysts, the tumor was colorless or blue, clear, round, soft and the surface was wet (Fig. 2C and D). In the two cases of heterotopic pancreas, the tumors were yellow, cloudy, soft and the tumor surfaces exhibited small nodules with an appearance similar to that of the pancreatic tissue itself or showing pancreatic-like tissue characteristics. (Fig. 4C). In the single case of lipoma, the tumor was yellow, clear, round, soft and the tumor surface appeared to be fatty and adipose tissue-like (Fig. 2B).

### Assessment II

The results of the statistical analysis of the comparison between the GIST and BST groups with regards to the five EVF are summarized in Table IV. Significant differences were identified between the GIST and BST groups in terms of the frequency of white (100% [13/13] vs. 61.5% [8/13]), cloudy (100% [13/13] vs. 53.8% [7/13]) and rigid tumors (100% [13/13] vs. 61.5% [8/13]; P<0.05 for all three), respectively. No significant differences were identified between the GIST and BST groups in terms of the frequency of nodular tumors (7.7% [1/13] vs. 15.4% [2/13]) and tumors with visible coatings (38.5% [5/13] vs. 23.1% [3/13]; P>0.05 for the two). Additionally, significant differences were observed between the two groups regarding the frequency of tumors with the combination of three EVF (white, cloudy and rigid), which was demonstrated in all 13 GISTs (100% [13/13] vs. 30.8% [4/13]; P<0.05 for the two).

## Discussion

SMTs are non-epithelial tumors that are covered by a normal mucosa. Unlike epithelial tumors, the majority of SMTs are endoscopically visualized as masses that protrude into the GI lumen. Thus, it is difficult to morphologically distinguish between the different types of SMT. Our novel tissue sampling method, i.e. a core biopsy using the SEMF method, enables a reliable histological diagnosis and the visualization of the tumor surface under endoscopic direct vision. This provides EVF of SMTs in the SM via a dissected submucosal tunnel, which can be assessed to differentiate between SMTs. To the best of our knowledge, this is the first report to characterize EVF of each type of SMT, particularly of GISTs, a type of SMT that is considered to possess malignant features. Therefore, the characteristic EVF may have potential diagnostic value for the differentiation of GISTs from other BSTs.

EUS is widely used for characterizing SMTs, and the information obtained by EUS, such as the layer from which an SMT arises, echogenicity and the internal structure of the tumor, enables the differential diagnosis between different types of SMT with a certain level of accuracy (15,16). For example, GISTs typically appear as a hypoechoic mass arising from the fourth hypoechoic GI wall layer (i.e. the MP) (17–20).

GISTs with malignant potential are significant for the differentiation from leiomyomas during diagnosis. Leiomyomas characteristically arise from the MP and are hypoechoic and homogeneous in their internal structures (17,18). Thus, it is difficult to distinguish GISTs from leiomyomas based only on the homogeneity of their internal structures. Furthermore, a substantial proportion of SMT cases exhibiting a hyperechoic submucosal layer include lipomas or heterotopic pancreas (3,17,18), which also cannot be reliably diagnosed by EUS. Thus, EUS should only be used as a supplementary diagnostic tool for determining the treatment strategy for SMTs and it is not intended to replace direct tissue sampling for the definitive diagnosis of SMTs. However, EUS-guided fine needle aspiration and tissue sampling procedures using the ESD technique have been shown to provide limited benefits (21–24). Furthermore, we have previously reported on the suitability of core biopsy using the SEMF method as a novel tissue sampling technique (13,14); in the present study, this technique provided accurate diagnoses in all cases.

According to Assessment I in the present study, and based on EVF obtained by core biopsy using the SEMF method, the endoscopic characteristics of the different types of SMTs may be summarized, which produces a novel classification of SMTs (Table III). Typical EVF are as follows: i) Low risk gastric GISTs, white, cloudy, round and rigid; ii) leiomyomas, white, almost clear, elastic hard tumors; iii) granular cell tumors and schwannomas, white, cloudy, round, rigid tumors comparable with GISTs (it is considered to be difficult to distinguish these tumors from GISTs using EVF); iv) gastric cysts, colorless or blue, clear, round, soft tumors with wet surfaces; v) heterotopic pancreas, yellow and small-nodular tumors. Among the SMTs, only heterotopic pancreases revealed a specific tumor surface with small nodules, which was characteristic of pancreatic tissues (25); and vi) lipomas, yellow, clear, soft tumors with adipose tissue-like characteristics.

According to Assessment II, each of the five EVF between the GIST and BST groups were compared; white tumors were observed in all of the 13 GIST cases, the five leiomyomas, the two granular cell tumors and the schwannoma. The two cases of heterotopic pancreas and the single case of lipoma presented yellow tumors, and the two cases of cysts demonstrated colorless or blue tumors. Although it is difficult to distinguish GISTs and benign tumors, including leiomyomas, granular cell tumors and schwannoma using color differences, it was possible to differentiate white GISTs from non-white benign tumors, such as heterotopic pancreas, lipoma and cysts. Furthermore, significant differences were observed with regards to clarity between the two groups. Specifically, GISTs and leiomyomas exhibited a difference with regard to clarity (0% [0/13] vs. 80% [4/5]). Thus, the clarity of the tumor surface between EVF may become an important index for distinguishing between GISTs and leiomyomas. The clarity of the tumor surface is considered to reflect the components and heterogeneity of its internal structures. These may be histological, and associated with the density of spindle cells and hyaline degeneration. Notably, the cystic tumor contained a fluid compartment, which may have contributed to its glossy and wet appearance. The association between EVF of the tumor surface and pathological characteristics will be investigated in our future studies.

With regard to the shape of the tumor surface, a large nodule was identified in one case (case 22: Tumor size, 32 mm; low risk GIST) of the 13 GIST cases, demonstrated that certain GISTs >2 cm exhibit nodular features compared with the 10 small GISTs (<2 cm in size), which had round surfaces (10/10 small GISTs). Small nodules were observed in only two of the cases of heterotopic pancreas among all of the SMTs. Therefore, the evaluation of the presence or absence of nodules may facilitate the distinction of specific tumors among SMTs. Visible tumor coatings were observed in 38.5% of GISTs (5/13) and in 23.1% of BSTs (3/13); the leiomyoma, schwannoma and the cyst. GISTs are generally encapsulated tumors, however, eight of the 13 GIST cases were not visually identified to have a thick capsule when observed under direct endoscopic view, indicating the limited diagnostic potential for the visual identification of a capsule.

Regarding solidity, there were significant differences identified between the two groups, indicating GISTs exhibit rigid tumors when compared with BSTs (100% [13/13] vs. 61.5% [8/13], respectively). Furthermore, concerning elastic or non-elastic tumors, all five leiomyomas presented with the feature of elastic hard tumors when compared with GISTs (100% [5/5] vs. 53.8% [7/13], respectively). Whether the mass indents, when pressure is applied using biopsy forceps, is commonly used for assessing the hardness of an SMT. GISTs and leiomyomas generally do not indent, which complicates the endoscopic differentiation of the tumors. Conversely, the elasticity of the mass, as obtained by core biopsy using the SEMF method, enables the assessment of the solidity of the tumor itself. This EVF provided the novel information that gastric leiomyomas are characteristically an elastic hard tumor.

There were statistically significant differences identified between GISTs and BSTs with regard to three EVF: Color, clarity and solidity. In addition, significant differences were observed between the two groups regarding the frequency of tumors that exhibited the combination of three specific EVF: White, cloudy and rigid, which were observed in all 13 GISTs, indicating that this combination of three EVF may be a useful parameter for differentiating between GISTs and BSTs.

With regard to clinical implications, a combination of TBB and visualizing the tumor surface, i.e. EVF, may be beneficial. This combination may aid with the decision as to whether the tumor requires resection. With an increasing number of reports describing the curative endoscopic resection of SMTs by ESD (26,27), further advances in diagnostics are required. Therefore, if the application of EVF assists with the diagnosis of SMTs, unnecessary and invasive resections may be avoided. The continued efforts to evaluate the clinical advantages of the current diagnostic techniques are anticipated to contribute to the development of novel criteria for diagnosing SMTs based on EVF. Finally, further studies are required to validate the specificity of this novel differential diagnostic approach. A prospective study to clarify the clinical application of EVF is currently ongoing.

In conclusion, gastric SMTs may be classified based on five EVF as follows: Color, clarity, shape, tumor coating and solidity, which indicates that EVF may possess potential diagnostic value for differentiating GISTs from BSTs.

## Figures and Tables

**Table I:** Clinicopathological data of patients with submucosal tumors.

Case	Age/Gender	Tumor size, mm	Layer	Echoic	Pathology
1	74/M	20	MP	Hypo	GIST, low risk
2	63/M	20	MP	Hypo	GIST, low risk
3	77/M	45	MP	Hypo	GIST, low risk
4	53/F	12	MP	Hypo	Leiomyoma
5	71/F	15	MP	Hypo	GIST, low risk
6	66/F	15	MP	Hyper	Heterotopic pancreas
7	76/F	15	MP	Hypo	GIST, low risk
8	55/F	20	MP	Hypo	GIST, low risk
9	82/F	15	MP	Hypo	GIST, low risk
10	51/F	15	MP	Hypo	Leiomyoma
11	75/F	25	SM	Hyper	Lipoma
12	67/F	12	MP	Hypo	GIST, low risk
13	56/M	25	SM	Anechoic	Gastric cyst
14	73/F	22	MP	Hypo	Schwannoma
15	62/M	15	MP	Hypo	Leiomyoma
16	49/F	15	SM	Hypo	Heterotopic pancreas
17	41/M	14	MP	Hypo	GIST, low risk
18	63/M	14	MP	Hypo	Granular cell tumor
19	63/M	8	MP	Hypo	Granular cell tumor
20	62/F	26	SM	Anechoic	Gastric cyst
21	63/M	22	MP	Hypo	GIST, low risk
22	63/F	32	MP	Hypo	GIST, low risk
23	72/F	14	MP	Hypo	GIST, low risk
24	82/F	13	MP	Hypo	GIST, low risk
25	53/M	15	MP	Hypo	Leiomyoma
26	54/F	22	MP	Hypo	Leiomyoma

M, male; F, female; MP, muscularis propria; GIST, gastrointestinal stromal tumor; SM, submucosa.

**Table II:** Five selective characteristic EVF findings of SMTs.

		Color				Clarity		Shape		Tumor coating		Solidity	
						
SMT	n	White	Blue	Colorless	Yellow	Clear	Cloudy	Round	Nodular	Visible	Not visible	Rigid	Soft
GIST, low risk	13	13	-	-	-	-	13	12	1 (Large)	5	8	13 (7 E, 6 NE)	-
Leiomyoma	5	5	-	-	-	4	1	5	-	1	4	5 (5 E)	-
Granular cell tumor	2	2	-	-	-	-	2	2	-	-	2	2 (2 E) -	
Schwannoma	1	1	-	-	-	-	1	1	-	1	-	1 (1 NE)	-
Cyst	2	-	1	1	-	2	-	2	-	1	1	-	2
Heterotopic pancreas	2	-	-	-	2	-	2	-	2 (Small)	-	2	-	2
Lipoma	1	-	-	-	1	1	-	1	-	-	1	-	1

EVF, endoscopically visualized features; SMT, submucosal tumor; GIST, gastrointestinal stromal tumor; E, elastic; NE, non-elastic.

**Table III:** Classification by EVF findings of gastric SMTs (proposed as a result of the present study).

SMT	EVF findings of gastric SMTs
GIST, low risk	White, cloudy, round or nodular, rigid
Granular cell tumor	White, cloudy, round, rigid
Schwannoma	White, cloudy, round, rigid
Leiomyoma	White, clear > cloudy, round, rigid (elastic hard)
Cyst	Blue or colorless, clear, round, soft
Heterotopic pancreas	Yellow, cloudy, small nodular, soft
Lipoma	Yellow, clear, round, soft

EVF, endoscopically visualized feature; SMT, submucosal tumor; GIST, gastrointestinal stromal tumor.

**Table IV:** Statistical analysis between the GIST and BST groups with regard to the five selective characteristic EVF findings and the combination of the three EVF findings (white, cloudy and rigid).

Characteristic	GIST, n=13 (%)	BST, n=13 (%)	P-value^a^
White color	13 (100)	8 (61.5)	0.039
Cloudy	13 (100)	7 (53.8)	0.014
Nodule	1 (7.7)	2 (15.4)	1.000
Visible coating	5 (38.5)	3 (23.1)	0.673
Rigid	13 (100)	8 (61.5)	0.014
Three EVFs (White, cloudy, rigid)	13 (100)	4 (30.8)	0.0005

a Fisher’s exact test (two-sided); P<0.05 indicates a statistically significant difference.

GIST, gastrointestinal stromal tumor; BST, benign submucosal tumor; EVF, endoscopically visualized feature.
